# Polysaccharide utilization loci encoded DUF1735 likely functions as membrane‐bound spacer for carbohydrate active enzymes

**DOI:** 10.1002/2211-5463.13816

**Published:** 2024-05-12

**Authors:** Lisanne Hameleers, Lucie A. Gaenssle, Salvador Bertran‐Llorens, Tjaard Pijning, Edita Jurak

**Affiliations:** ^1^ Department of Bioproduct Engineering University of Groningen The Netherlands; ^2^ Department of Chemical Engineering University of Groningen The Netherlands; ^3^ Department of Biomolecular X‐ray Crystallography, Groningen Biomolecular Sciences and Biotechnology Institute (GBB) University of Groningen The Netherlands

**Keywords:** Bacteroidetes‐Associated Carbohydrate‐binding often N‐terminal (BACON) domain, domain analyzer program, domain of unknown function 1735 (DUF1735), N‐terminal spacer, polysaccharide utilization loci (PULs), vicinity analyzer program

## Abstract

Proteins featuring the Domain of Unknown Function 1735 are frequently found in Polysaccharide Utilization Loci, yet their role remains unknown. The domain and vicinity analyzer programs we developed mine the Kyoto Encyclopedia of Genes and Genomes and UniProt to enhance the functional prediction of DUF1735. Our datasets confirmed the exclusive presence of DUF1735 in Bacteroidota genomes, with *Bacteroidetes thetaiotaomicron* harboring 46 copies. Notably, 97.8% of DUF1735 are encoded in PULs, and 89% are N‐termini of multimodular proteins featuring C‐termini like Laminin_G_3, F5/8‐typeC, and GH18 domains. Predominantly possessing a predicted lipoprotein signal peptide and sharing an immunoglobulin‐like β‐sandwich fold with the BACON domain and the N‐termini of SusE/F, DUF1735 likely functions as N‐terminal, membrane‐bound spacer for diverse C‐termini involved in PUL‐mediated carbohydrate utilization.

## Graphical abstract

DUF1735 are found in Bacteroidota, particularly within Polysaccharide Utilization Loci (PULs). Data generated with our domain and vicinity analyzer programs, identified DUF1735 as N‐termini of multimodular proteins featuring C‐termini like Laminin_G_3, F5/8‐typeC, or GH domains, with an Ig‐like β‐sandwich fold. A function as a membrane‐bound spacer for varying C‐termini, potentially involved in PUL‐mediated carbohydrate utilization, is proposed.

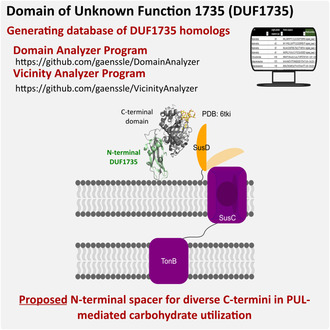

**Abbreviations**
BACON: Bacteroidetes‐Associated Carbohydrate‐binding often N‐terminal (IPR024361, PF13004, cd14948)
CAZyme: Carbohydrate Active enZyme
CBM: Carbohydrate Biding Module
DUF: Domain of Unknown Function
F5/8‐typeC: also known as discoidin domain family (IPR000421, PF00754)
GH: Glycoside Hydrolase
HGM: Human Gut Microbiota
Ig: Immunoglobulin
KEGG: Kyoto Encyclopedia of Genes and Genomes
KO: KEGG Orthology
LamG3: Lamninin_Globular_3 (IPR001791)
LRR_5: Leucine‐Rich Repeat_5
PDB: Protein Data Bank
PeSTo: ProtEin STructure transfOrmer
Por_secre_tail: Por secretion system C‐terminal sorting domain
PUF: Protein of Unknown Function
PUL: Polysaccharide Utilization Locus
PULDB: Polysaccharide Utilization Loci DataBase
RagB: SusD‐associated domain
Sulf: Sulfatases
Sus: Starch utilization system
SusD‐D: SusD‐associated domains ‘SusD‐like domain’ or ‘RagB’
SusD‐ID: domains annotated as SusD KO (K21572)
SusD‐N: domains annotated as ‘SusD’ and ‘RagB’

For the discovery of Carbohydrate Active enZyme (CAZyme), bacterial genomes are often investigated because they encode highly efficient enzymatic machineries that enable the host to degrade carbohydrates used for glycolysis [1, 2, 3, 4, 5]. One machinery studied in detail is the Starch utilization system (Sus) of *Bacteroides thetaiotaomicron* [6, 7, 8, 9, 10, 11, 12]. It consists of eight proteins (SusR, A‐G) required to sense, bind, transport, and degrade starch. All eight proteins are encoded in a physically linked gene cluster with co‐regulated expression (Fig. 1A). Upon maltose binding, the inner membrane‐spanning transcriptional regulator SusR induces the expression of the *susA‐G* gene cluster (Fig. 1B) [12]. Subsequently, the lipid‐anchored sugar‐binding protein SusD and the cell surface starch‐binding proteins SusE and SusF, bind starch and provide local proximity between the substrate and the membrane‐bound *endo*‐acting α‐amylase SusG [6, 7, 8, 9, 10, 11]. SusG further hydrolyses starch into maltooligosaccharides, which are transported into the periplasmic space via the TonB‐dependent sugar transporter SusC. In the periplasm, SusA and SusB, acting as neopullulanase and α‐glucosidase respectively, further degrade the maltooligosaccharides into glucose, which is transported into the cell for glycolysis (Fig. 1B). Similar physically linked gene clusters encoding synergistically acting proteins to degrade complex carbohydrates were found to be conserved within the phylum of Bacteroidota (previously named Bacteroidetes) [4, 13, 14, 15, 16]. Since the *susC/D* gene pair, encoding SusC/D, was highly conserved, its homologs became the genetic markers of gene clusters named Polysaccharide Utilization Loci (PULs). The PUL database (PULDB) (www.cazy.org/PULDB/ [17]) predicts PULs in sequenced genomes by localizing a *susC/D* gene pair and extending the PUL with physically linked genes encoding CAZymes and regulatory proteins. Besides proteins with predicted functions, PULs can also contain Proteins of Unknown Function (PUFs) that often contain Domains of Unknown Function (DUFs), making PULDB a valuable tool for novel CAZyme discovery. A powerful advantage of PUL‐based enzyme discovery is the possibility to predict putative substrates for present PUFs by matching the predicted catalytic functions of annotated enzymes of the PUL with monosaccharides and linkages present in a substrate.

**Fig. 1:**
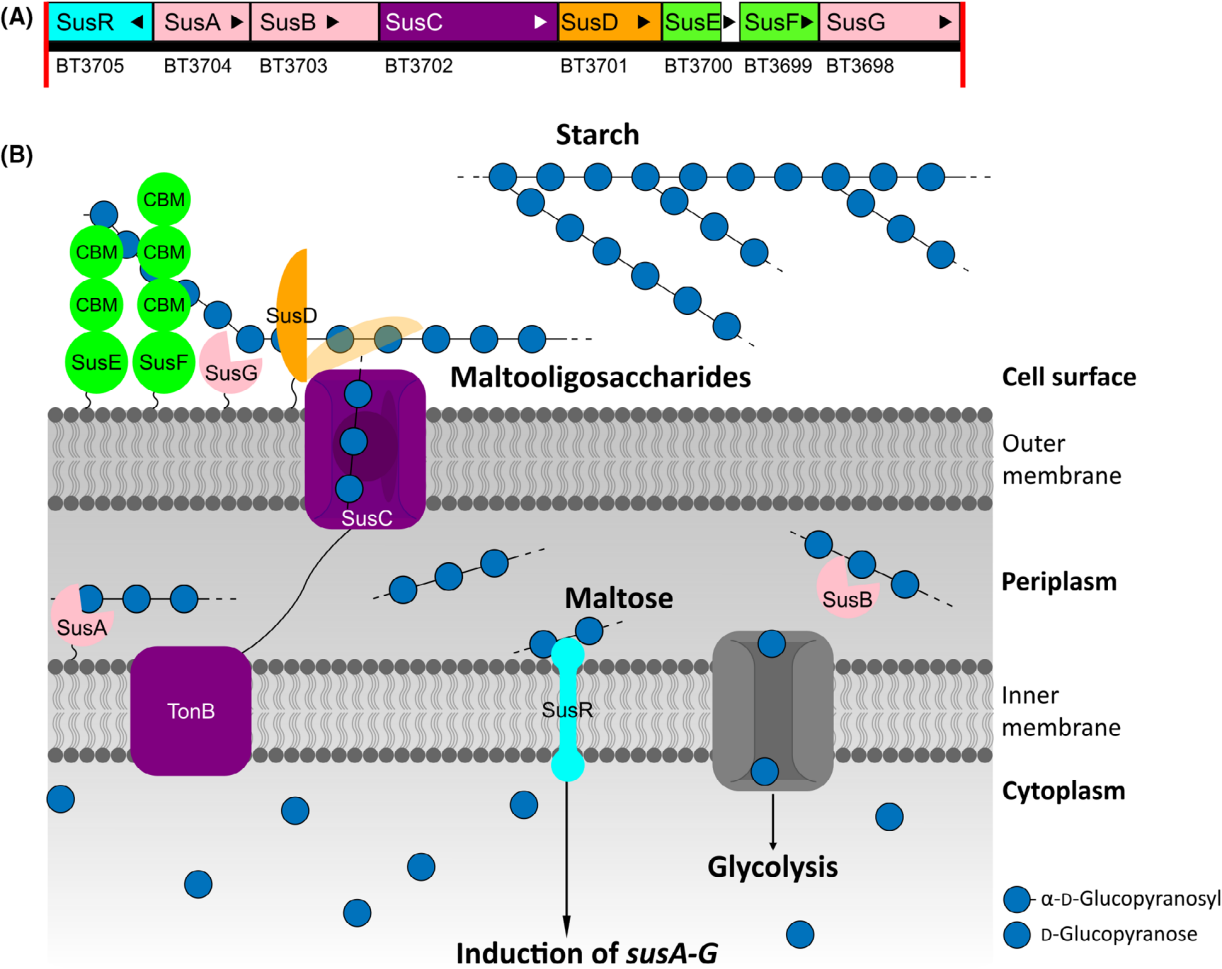
Scheme of the Starch utilization system (Sus) in *Bacteroides thetaiotaomicron* based on [1, 14, 58]. (A) Scheme of Sus gene cluster consisting of BT3698‐BT3705 (annotated as PUL66 in PULDB (www.cazy.org/PULDB/ [17])). Gene sizes are represented to scale; red bars indicate the margins of assembled region. (B) The transcription regulator SusR induces transcription of *susA‐G* genes upon maltose binding. SusA‐G proteins work synergistically for efficient starch degradation. The three starch‐binding outer membrane lipoproteins SusD‐F bind starch, bringing the substrate in close proximity to the membrane‐bound α‐amylase SusG. SusG hydrolyses starch into maltooligosaccharides that transit into the periplasm through the Ton‐B dependent transporter SusC. In the periplasm, the neopullulanase SusA and the α‐glucosidase SusB hydrolyze maltooligosaccharides into glucose that is transported into the cytoplasm for glycolysis.

While using this approach, PUFs containing DUF1735 have been observed frequently one to two genes downstream of the *susC/D* gene pair (referred to as the *susE‐F* position). Literature reported that DUF1735 was the most overrepresented Pfam‐A domain (curated Pfam domains; automatically generated domains are labeled as Pfam‐B) in the Human Gut Microbiota (HGM) and a remote homology of DUF1735 to SusE was mentioned [18, 19, 20]. To our knowledge, only two DUF1735‐containing proteins, namely BT3986 (DUF1735 + LamG3; UniProt: Q8A0N5_BACTN) and BT3987 (DUF1735 + GH18; UniProt: Q8A0N4_BACTN) have been investigated but only their C‐termini were functionally characterized and used for naming the complete protein [21, 22, 23]. The exclusion of DUF1735 in the protein annotation made gathering all published knowledge challenging. Therefore first, PULDB was used to identify all literature‐derived PULs encoding DUF1735 to collect their corresponding literature. Subsequently, we developed two programs, the domain and vicinity analyzer that generated comprehensive datasets of all DUF1735‐containing proteins available in the Kyoto Encyclopedia of Genes and Genomes (KEGG) and UniProt [23, 24]. This dataset allowed the analysis of the taxonomical distribution of DUF1735 homologs, their abundance within genomes, as well as their PUL and domain architecture.

Due the observed abundance and specific location of DUF1735 at *susE‐F* position in PULs of species belonging to the Bacteroidota phylum, we hypothesized that DUF1735 likely has a function in bacterial carbohydrate utilization. The previously investigated Bacteroidota‐Associated Carbohydrate‐binding Often N‐terminal (BACON) domain (IPR024361, PF13004, cd14948) was also frequently found in the phylum of Bacteroidota and was described as N‐terminal domains of multimodular proteins involved in carbohydrate utilization and mucin was predicted as putative substrate [6, 13, 15, 16, 25]. The viral‐specific crAss‐BACON domain (from now on called BACON_2, PF19190) evolved from the BACON domain and is encoded in crAss‐like phage that are predicted to infect species of the Bacteroidota phylum [25, 26]. The common N‐terminal location and association to the Bacteroidota phylum of DUF1735, BACON, and BACON_2 motivated us to generate the same dataset for all three domains [25, 26].

## Materials and methods

### Analysis of DUF1735‐containing proteins within literature‐derived PULs

The locus tags of DUF1735 (PF08522)‐containing proteins within literature‐derived PULs in PULDB (www.cazy.org/PULDB/ [17]) were provided by Prof Nicolas Terrapon (accessed on 15.09.2022). For each hit the gene ID, host species, domain architecture, predicted functional annotation, and predicted substrate specificity of the PUL were manually extracted from PULDB and a database was created (Table S1). This initial dataset was used to confirm its abundance in PULs and to determine the position of DUF1735‐containing proteins within literature‐derived PULs.

### Mining KEGG and UniProt for homologs with the domain analyzer

The automated analysis of the target domains was conducted with the domain analyzer (https://github.com/gaenssle/DomainAnalyzer), a program written in python 3.8 [27]. This program performs three distinct steps. First, it retrieves all gene IDs associated with the entered domain name (DUF1735, BACON, BACON_2) from the Kyoto Encyclopedia of Genes and Genomes (KEGG) and UniProt [23, 24] via the DB GET function of genome.jp. Then, information on taxonomy, sequence, and domain architecture are downloaded for each gene ID. For UniProt IDs, data is retrieved via genome.jp. KEGG entries are obtained using the KEGG package from the Biopython module (Bio.KEGG.REST) with exception of the functional domain annotation, which was accessed from kegg.jp and subsequently filtered with a cutoff E‐Value of 0.0001 to refine the analysis of the domain architecture. Last, the retrieved data are summarized and counted based on the distribution of domain architecture and taxonomic classification. The generated datasets are available in Tables S2A–C and S3 and are based on data downloaded in February 2024.

### Studying genomic neighbors of target domains with vicinity analyzer

To study whether a target domain (DUF1735, BACON, or BACON_2) was encoded in a PUL, the vicinity analyzer (https://github.com/gaenssle/VicinityAnalyzer) was developed. It counts the occurrence of SusD (or other inputs) in ±5 gene distance of the target domain. The continuous numeration of genes within a genome stored in KEGG was exploited, as KEGG gene IDs consist of the corresponding KEGG genome ID and the index of the gene within the genome. As the enumeration of the gene indices for each genome were not always adjacent numbers (e.g. 1, 2, 3, …) but also occurred in steps of 5 and 10, the program was written to both detect and correct for such index numbering. By calculating the distance between KEGG gene IDs neighboring genes can be identified. By calculating the distance between KEGG gene IDs neighboring genes can be identified. SusD was selected as reliable PUL marker because it was consistently assigned a KEGG Orthology (KO). Besides the SusD KO K21572 (indicated as SusD‐ID in Table S4), the SusD‐associated domains ‘RagB’ and ‘SusD‐like domain’ (indicated as SusD‐D in Table S4), and the corresponding annotations ‘SusD’ and ‘RagB’ (indicated as SusD‐N in Table S4) were applied as indirect PUL identifier. The generated dataset is based on data downloaded in February 2024 and can be found in Table S4.

### Analyzing cellular localization based on signal peptide prediction

The extracted protein sequences of DUF1735‐, BACON‐, and BACON_2 domain‐containing proteins (Table S2A–C, FASTA files on request) were used as input for signal peptides prediction using signalp6.0 [28].

### Conserved residues and their structural localization in DUF1735‐containig proteins

The Multiple Sequence Alignments of DUF1735, BACON, and BACON_2 seed sequences were downloaded from InterPro (accessed in February 2024) [29]. BT3987 (6T8i) was analyzed with ConSurf and the pre‐calculated ConSurfDB analysis was used [30, 31].

### PeSTo analysis

DUF1735 crystal structures deposited in PDB were analyzed to predict interactions of the protein with other proteins, DNA/RNA, lipids, ligands, and ions using PeSTo (https://pesto.epfl.ch/) [32].

## Results and Discussion

### Analysis of DUF1735‐containing proteins in literature‐derived PULs

While mining PULs for CAZyme discovery, DUF1735‐containing proteins were frequently observed at the *susE‐F*‐like position in the PULs. To quantitatively confirm this observation and obtain more relevant literature, a dataset consisting of all literature‐derived PULs in PULDB that contain one or more DUF1735 was created. A total of 135 individual DUF1735 domains, 117 DUF1735‐containing proteins being encoded in 86 literature‐derived PULs of 11 different species all belonging to the Bacteroidota phylum, and 10 corresponding publications were collected (Table S1) [33, 34, 35, 36, 37, 38, 39, 40, 41]. Manual analysis of the obtained dataset confirmed that all DUF1735‐containing proteins were indeed exclusively encoded at the *susE*‐like (47%) or *susF*‐like (35%) position, and only occasionally at *sus‐G*‐like position (18%; Table S1). Besides the DUF1735‐containing proteins the most commonly predicted CAZymes in the 86 DUF1735‐containing literature‐derived PULs were the SusC/D markers (11% each), and members of the Glycoside Hydrolase family 18 (GH18; 6%), GH92 (4%), GH130 (1%), GH2 (1%), and Sulfatases family 1 (Sulf_1; 1.5%; Table 1). Members of GH18 are described as enzymes capable of chitin degradation or non‐catalytic proteins such as xylanase inhibitors [42], concanavalin B [43], and narbonin [44]. Members of GH92 are enzymes involved in the degradation of mannose‐containing carbohydrates [21, 45] and members of GH130 are mannose specific phosphorylases [46]. GH2 is a very diverse family of predominantly *exo*‐acting hydrolases accepting an array of different monosaccharides among which mannose [47]. Thus, although the function of the majority (32% PUFs) of all proteins encoded in DUF1735‐containing literature‐derived PULs was unknown, mannose‐based carbohydrates were the best informed suggestion as possible targets of the proteins encoded in these PULs. This was further supported by the *endo*‐β‐*N*‐acetylglucosaminidase activity of BT3987 (DUF1735 + GH18), the only DUF1735‐containing protein with described catalytic function to date [21]. BT3987 is encoded in PUL72 (referred to as HMNG‐PUL) of *Bacteroidetes thetaiotaomicron* VPI‐5482 and it degrades high‐mannose mammalian *N*‐glycan (HMNG) together with the other enzymes of the PUL, including the sugar‐binding protein BT3986 (DUF1735 + LamG3) another DUF1735‐containing protein (discussed later) [21].

**Table 1:** Annotations of Carbohydrate Active enZymes (CAZymes) given to all 1414 proteins encoded in 86 DUF1735‐containing literature‐derived Polysaccharide Utilization Loci (PULs) extracted from PULDB (www.cazy.org/PULDB/ [17]). For more information and specific references, see Table S1.

CAZy annotation	Function	[*n*]	[%]
PUF	Protein of Unknown Function	449	31.8
SusD	SusD‐like outer membrane‐binding protein	160	11.3
SusC	SusC‐like TonB‐dependent transporter	159	11.2
GH18	Glycoside Hydrolase family 18	83	5.9
Extracytoplasmic function sigma factor (ECF‐σ)	Regulator	68	4.8
Extracytoplasmic function anti‐sigma factor (Anti‐σ)	Regulator	65	4.6
GH92	Glycoside Hydrolase family 92	62	4.4
Hybrid two‐component system (HTCS)	Regulator	34	2.4
Sulf_1	Sulfatase	21	1.5
GH130	Glycoside Hydrolase family 130	19	1.3
GH2	Glycoside Hydrolase family 2	18	1.3
Major facilitator family transporter (MFS)	Transporter	15	1.1
Other (< 15 entries)		261	18.5

Our initial screening for DUF1735‐containing proteins in literature‐derived PULs confirmed their conserved location at *susE‐G*‐like position within PULs and suggested their relevance for the phylum of Bacteroidota whose species are known to produce a vast amount of enzymes required for complex carbohydrate degradation [4, 13, 14, 15, 16].

### Taxonomic distribution and genomic localization of DUF1735‐homologs

**Fig. 2:**
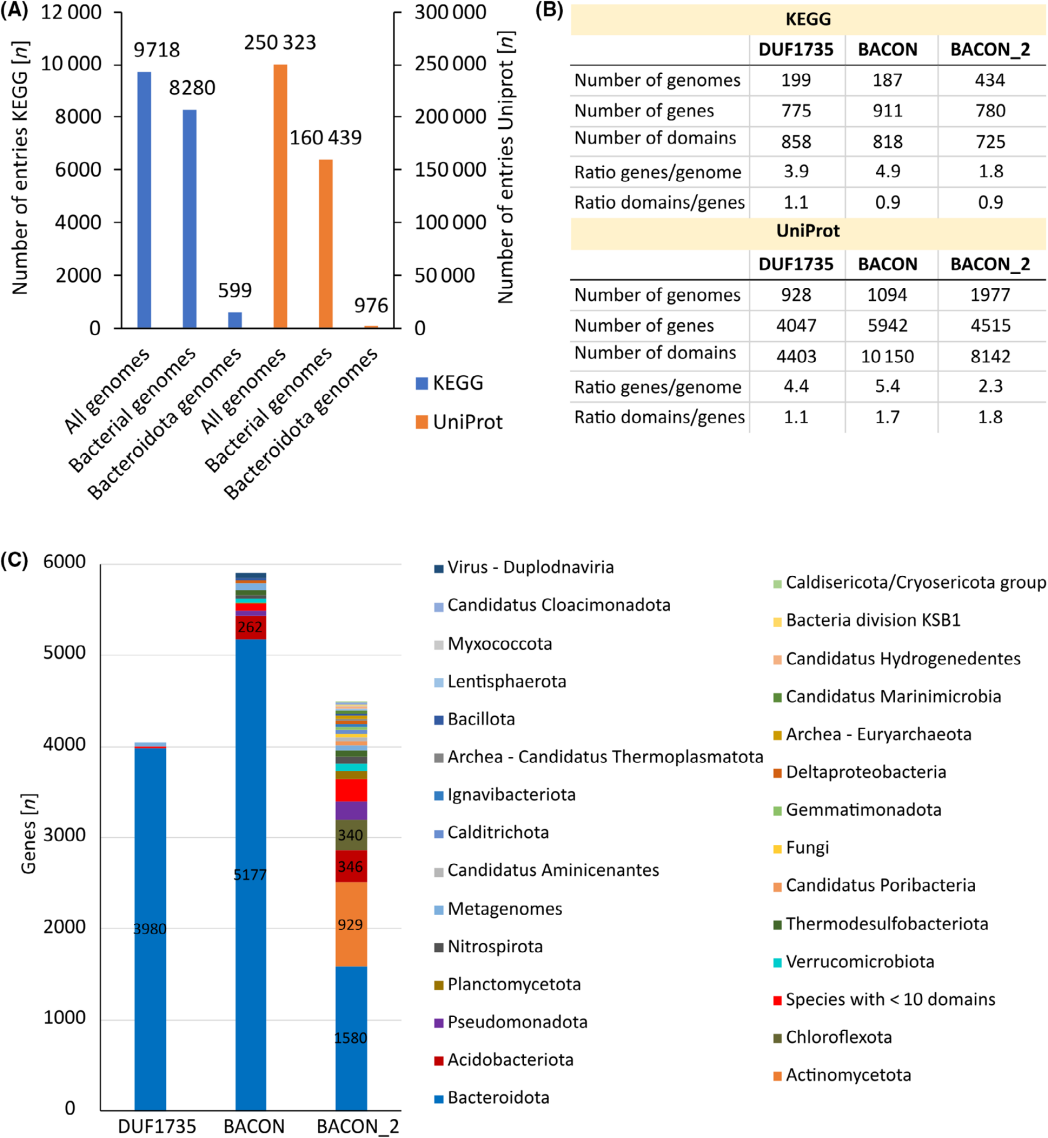
Quantification and taxonomic distribution of DUF1735, BACON, and BACON_2 domains within genomes in KEGG and Uniprot [23, 24]. (A) Quantification of total, bacterial, and Bacteroidota genomes. (B) Quantification of genomes and genes encoding the target domains, as well as quantification of individual target domains. (C) Taxonomic phyla distribution of the target domain in UniProt.

To provide a comprehensive overview of DUF1735‐containing proteins beyond literature‐derived PULs, KEGG and UniProt were mined for DUF1735 homologs by using the domain analyzer, a newly developed program. KEGG contained 9718 genomes of all kingdoms of life (Plants, Fungi, Animals, Bacteria, Archaea, and Protista) of which the majority (85%) was of bacterial origin (Fig. 2A). UniProt is the bigger database with 250 323 total genomes out of which 68% are bacterial (Fig. 2A). The extended dataset of DUF1735 homologs increased the number of individual DUF1735 domains by 5.7‐fold (KEGG) and 30‐fold (UniProt) from 135 found in literature‐derived PULs to 775 in KEGG and 4047 in UniProt (Fig. 2B, Table S2A). For BACON and BACON_2 domains 911 and 780 entries in KEGG and 5942 and 4515 entries in UniProt were identified, respectively (Fig. 2B, Table S2B,C). For each entry, the KEGG genome and gene ID, the Uniprot ID, its annotation, its kingdom, phyla, and species of origin, the protein sequence and length, as well as its domain architecture were collected (Table S2A–C). To investigate whether the extended list of DUF1735 homologs were consistently encoded in PULs their vicinity to a SusD homolog was determined by using the vicinity analyzer and the KEGG dataset. The results revealed that the majority (98%) of DUF1735 homologs were indeed in close vicinity to a SusD homolog, out of which 14% were identified via its unique KO K21572 (Tables S4 and S5). This exclusive presence of DUF1735‐containing proteins in PULs suggests their involvement in PUL‐based carbohydrate utilization. In comparison, only 38% of BACON domains and 28% of BACON_2 domains were found to be encoded in PULs (Tables S4 and S5).

Interestingly, of all bacterial genomes in UniProt only 0.6% belong to the Bacteroidota phylum (Fig. 2A) and 98% of all DUF1735 are encoded in one of their genomes (Fig. 2C). The virtually exclusive presence of DUF1735 within Bacteroidota genomes and the fact that 85% of all Bacteroidota genomes contained at least one DUF1735, highlights its evolutionary importance for this phylum (Fig. 2A,B). Similar to DUF1735, also the majority of all BACON domain‐containing proteins (87%, 5177) were encoded in species of Bacteroidota (Fig. 2B,C). These results were as expected and aligned with the previously published correlation between BACON domains and the phylum of Bacteroidota [25]. The BACON_2 domain is also most frequently encoded in species of Bacteroidota but only 35% (1580, Fig. 2B,C). Notably, 16% (929) of all BACON_2‐containing proteins were found in the Gram‐positive phylum of Actinomycetota, highlighting a difference between DUF1735 and BACON_2 domain and making BACON_2 domains the only domains present in Gram‐positive species (Fig. 2C).

At least one copy of the 775 DUF1735 was present in 199 genomes resulting in an average of 4.0 DUF1735 per genome (Fig. 2B). A detailed analysis of the numbers of DUF1735 per genome, however, revealed that 13 species contained > 10 DUF1735 within their genomes with a maximum number of 46 DUF1735 in *B. thetaiotaomicron* 7330 (Table 2). This high number of gene copies suggests a relevant function of DUF1735 for *B. thetaiotaomicron* 7330, which is a keystone member of the HGM and a known specialist in carbohydrate degradation [48, 49]. All 46 DUF1735 were encoded in one of the many PULs of this species (Tables S2A and S4). Twelve of the species containing > 10 DUF1735 in their genome belonged to the order of *Bacteroidales* whose members are also commonly found in the HGM, while *Paraflavitalea soli* was isolated from greenhouse soil (Yongin city, Korea [50]) and belonged to the order of *Chitinophagales*.

**Table 2:** Overview of species containing > 10 DUF1735 in their genome. KEGG database was used due to unique identifier per sequenced genomes.

Species	DUF1735‐containing proteins [*n*]	DUF1735 domains [*n*]	*n* domains/*n* protein [mean]
*Bacteroides thetaiotaomicron 7330*	38	46	1.2
*Bacteroides thetaiotaomicron VPI‐5482*	34	39	1.1
*Bacteroides faecis*	30	35	1.2
*Bacteroides ovatus*	27	28	1.0
*Bacteroides faecium*	25	31	1.2
*Bacteroides xylanisolvens*	17	20	1.2
*Bacteroides finegoldii*	16	16	1.0
*Alistipes shahii*	14	16	1.1
*Alistipes onderdonkii subsp. vulgaris*	13	16	1.2
*Bacteroides caecimuris*	13	16	1.2
*Alistipes finegoldii*	12	14	1.2
*Bacteroides caccae*	10	16	1.6
*Paraflavitalea soli*	10	10	1.0
Other (< 10 entries)	516	555	1.1

The extended dataset generated by domain and vicinity analyzer confirmed the virtually exclusive presence of DUF1735 in PULs of species belonging to the Bacteroidota phylum with up to 46 DUF1735 domains in a single genome. These results further supported the hypothesis that DUF1735‐containing proteins are involved in PUL‐driven carbohydrate utilization.

### Domain architecture, N‐terminal membrane association, and review of functional characterization of DUF1735‐containing proteins

To obtain information about putative functions of DUF1735, the domain annotations of DUF1735‐containing proteins and their specific domain architecture were analyzed in the produced KEGG and UniProt dataset (Tables S2A–C and S3). The majority of DUF1735‐ (84.1%), BACON‐ (67.5%), and BACON_2 domain‐containing proteins (69.2%) were multimodular but their C‐termini differed (Table S2A–C). For DUF1735‐containing proteins 39 different C‐termini were identified with LamG3 (22.3%), F5/8‐typeC (20.7%), GH18 (5.9%), GH16 (0.5%) and GH43 (0.4%) being the most common, functionally characterized C‐termini, and DUF4361 (19.9%), DUF1735 (12.7%), and DUF5627 (10.2%) being the most common uncharacterized C‐termini (Table S2A). These results were in accordance with the initial dataset obtained from PULDB (Table S1). In the dataset obtained from UniProt, 16 different C‐termini were present including BT‐3044‐like C‐terminal that belongs to DUF4361, DUF5627, GH18, and F5/8‐typeC (Tabel S3) [51]. Interestingly, LamG3 was not represented (Table S3).

**Fig. 3:**
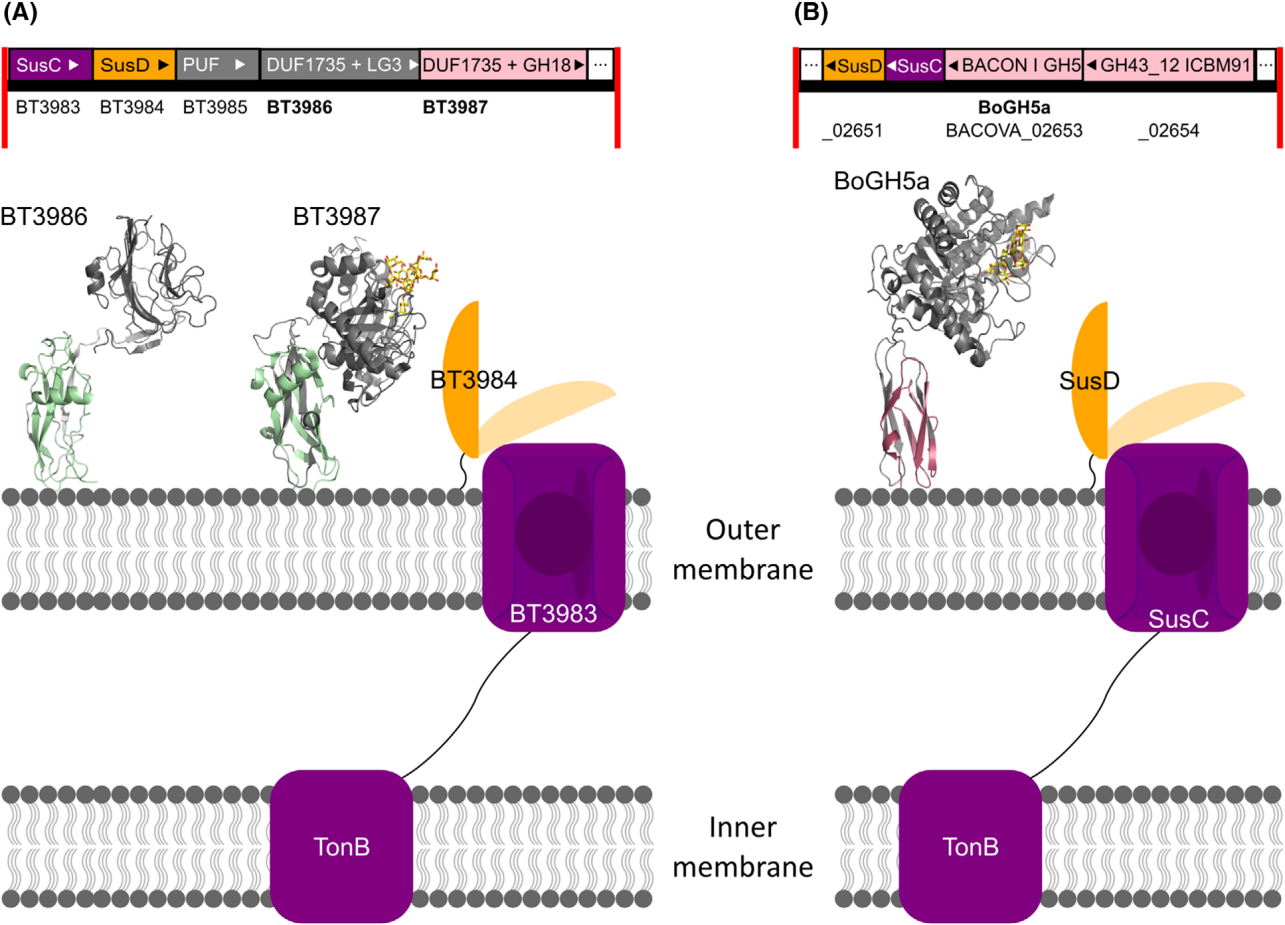
Schematic representation of expected cellular location of DUF1735‐ and BACON‐containing proteins. (A) alphafold model of BT3986 (green DUF1735 + gray LamG3; pLDDT = 88.4), and crystal structure of BT3987 (green DUF1735 + gray GH18; PDB 6t8k) with bound substrate Man9GlcNAc (yellow). (B) Crystal structure of BoGH5A (red BACON + gray GH5; PDB 3ZMR) with bound substrate Glc4Xyl3 (yellow). Location of BoGH5A was proposed in [53]. For all three proteins, a N‐terminal lipoprotein was predicted (not depicted) suggesting the proteins to be membrane‐bound.

The multimodular proteins had varying domain architectures. The most frequent and thus the most representative domain architecture for DUF1735‐containing proteins consists of an N‐terminal DUF1735 and a C‐terminal LamG3 domain (20.6%; Table 3). To our knowledge, only one protein consisting of DUF1735 + LamG3 has been functionally investigated, namely the previously mentioned protein BT3986 [21]. It is encoded in the literature‐derived PUL72 of human gut microorganism *B. thetaiotaomicron* VPI‐5482 and involved in the synergistic HMNG degradation together with enzymes [21]. PUL72 encodes BT3986 (DUF1735 + LamG3) at *susF*‐position and the previously mentioned BT3987 (DUF1735 + GH18) at *susG*‐position [21]. Further, it encodes three GH92, three PUFs, and two regulators (ECF‐σ and Anti‐σ; Fig. 3A) [21].

**Table 3:** Domain architectures of DUF1735‐containing proteins in KEGG and UniProt [23, 24]. *indicates that overlapping DUF1735IDUF4973 and DUF4973IDUF1735 are included. For more details see Tables S2A and S3.

KEGG (775 total)	[*n*]	[%]	UniProt (4047 total)	[*n*]	[%]
DUF1735* + LamG3	160	20.6	DUF1735	2249	55.6
DUF1735* + DUF4361	146	18.8	DUF1735 + BT‐3044‐like C‐terminal	593	14.7
DUF1735*	122	15.7	DUF1735 + DUF5627	497	12.3
DUF1735* + F5/8‐typeC	95	12.3	DUF1735 + DUF1735	231	5.7
DUF1735* + DUF5627	75	9.7	DUF1735 + GH18	163	4.0
DUF1735 + GH18	43	5.5	DUF1735 + F5/8‐typeC	88	2.2
DUF1735* + DUF1735 + F5/8‐typeC	43	5.5	DUF1735 + DUF1735 + F5/8‐typeC	80	2.0
DUF1735 + DUF1735	17	2.2	DUF1735 + GLUG	32	0.8
DUF1735 + LamG3IPentaxin	12	1.5	Other (≤ 20)	114	2.8
DUF4999 + DUF1735 + DUF1735 + F5/8‐typeC	5	0.6			
Other (*n* ≤ 5)	57	7.4			

Similar to the function of SusE or SusF, BT3986 (DUF1735 + LamG3) acted as surface‐bound sugar‐binding protein, keeping the produced oligosaccharides in close proximity to the cell surface (Figs 1 and 3A) [21]. The N‐terminal DUF1735 domain of BT3986 (DUF1735 + LamG3) encodes a lipoprotein signal peptide and is likely responsible for anchoring its C‐terminus to the cell surface (Fig. 3A). Notably, it was not specified which of the two domains had carbohydrate‐binding [21], however, it is likely that the C‐terminal LamG3 domain is responsible for it as carbohydrate binding capacities had been described previously for this domain [52]. The two LamG3 domains of the multimodular arabinofuranosidase Abf43A‐Abf43B‐Abf43C (WP_024834488) from *Ruminiclostridium josuimodular*, are examples. They were characterized as new Carbohydrate Binding Modules (CBMs) capable to recognize α‐(1,5)‐linked l‐arabinofuranosyl residues [52]. A predictive analysis of the protein binding interface of the alphafold model of BT3986 with PeSTo (https://pesto.epfl.ch/) further revealed that DUF1735 does not show any predicted interactions with ligands, while some residues of LamG3 were indicated to interact with ligands with prediction scores between 0.5 and 0.8 (Fig. S1).

Similar to the function of SusG, it has been shown that BT3987 (DUF1735 + GH18) acted as surface‐bound GH; specifically as *endo‐N*‐acetylglucosaminidase separating the oligosaccharide moiety in HMNG from its polypeptide (Figs 1 and 3A) [21]. Besides, Trastoy *et al*. solved five crystal structures of BT3987 (DUF1735 + GH18) in complex with its substrates and products, revealing that the N‐terminal DUF1735 is not involved in substrate binding, which has been confirmed by a PeSTo analysis [22, 32]. Thus, no catalytic activity or carbohydrate binding capacity for DUF1735 itself has yet been described but a membrane‐linker function that provides proximity of its C‐termini to other membrane‐bound enzymes is suggested. This was not far‐fetched as a similar function has been proposed for BACON domains [53]. BoGH5A (BACON + GH5) encoded in PUL113 of *B. ovatus* ATCC 8483 has been functionally characterized and similar to BT3987 (DUF1735 + GH18) it contains a non‐catalytic N‐terminal and a catalytic C‐terminal domain [21, 53]. The catalytic GH5 showed *endo*‐xyloglucanase and acted as keystone enzyme for PUL‐based xyloglucan degradation in *B. ovatus*. Based on its solved crystal structure (PDB: 3ZMR) and the predicted lipoprotein signal peptide in the BACON domain, the authors suggested that BACON anchors the catalytic C‐terminus to the membrane, thus, acting as spacer allowing GH5 to freely move close to the membrane (Fig. 3B) [53].

The second most abundant domain architecture for DUF1735‐containing proteins was DUF1735* + DUF4361 (18.8%, Table 3). Interestingly, DUF4973 is mostly overlapping with DUF1735 domain which might question their distinction (indicated as DUF1735*). Further, it was observed that DUF4973, DUF4361, and DUF5627 were exclusively present in combination with the N‐terminal DUF1735 (Table S6). To our knowledge, no protein containing these domains has been functionally characterized to date but in the previously mentioned study by Cuskin *et al*. [21], two PULs encoding each a multimodular protein consisting of an N‐terminal DUF1735 and a C‐terminal DUF4361 (BT2624, BT3790) were described. BT2624 and BT3790 were both encoded at the *susE*‐like position in the two mannan degrading PULs, MAN‐PUL1 (PUL 90 in PULDB) and MAN‐PUL2. While the functional characterization of BT2624 and BT3790 is yet to be determined, there is a strong likelihood that proteins containing both DUF1735 and DUF4361 play a significant role in mannan degradation in *B. thetaiotaomicron* due to their simultaneous upregulation and expression with other functional members of the PULs [12].

Another interesting domain architecture is DUF1735* + DUF5627 (9.7%, Table 3) which was observed in several previously investigated PULs whose encoded enzymes target xylan [35, 54, 55, 56, 57]. Despres *et al*. [54], described a core xylan utilization cluster within a PUL of *B. xylanosolvens* (BXY_29250‐BXY_29300) consisting of a double *susCD* gene pair followed by a PUF (DUF1735 + DUF5627) and a GH10. These genes were specifically upregulated if *B. xylanosolvens* was cultivated on xylan with respect to cultivation on glucose as carbon source [54]. This core xylan utilization cluster has been shown to be conserved throughout several *Bacteroidetes* strains suggesting a role of DUF1735‐containing proteins in xylan degradation [35, 54, 55, 56, 57].

For BACON‐ and BACON_2 domain‐containing proteins, the diversity of C‐termini was with 151 and 156 different domains, four‐times higher compared to DUF1735‐containing proteins, respectively (Table S2A–C). Similar to DUF1735 and DUF4973, BACON and BACON_2 domains frequently overlap indicating their similarity. They are, however, also the most frequent independent C‐termini for BACON and BACON_2 domain‐containing proteins (Table S2B,C). Other C‐terminal domains were F5/8‐typeC, cellulases, Leucine‐rich repeat_5 (LRR_5), and Por secretion system C‐terminal sorting domain (Por_secre_tail). Notably, LamG3 was only rarely present as C‐terminus for BACON, and BACON_2 domain‐containing proteins (Table S2B,C).

To investigate whether DUF1735‐, BACON‐, and BACON domain‐containing proteins were membrane‐bound, the extracted protein sequences were used as input for signalp6.0 to predict signal sequences (Tables S4 and S5) [28]. The most frequently predicted signal peptide for DUF1735‐ (96%) and BACON‐containing proteins (76%) encoded an N‐terminal lipoprotein signal peptide (Tables S4 and S5). A smaller fraction (1% of DUF1735, 10% of BACON, 25% of BACON_2) encoded an N‐terminal signal peptide for secretion or no signal peptide (4% of DUF1735, 14% of BACON, 0% of BACON_2). These results suggest that most DUF1735‐, and BACON domain‐containing proteins and a third of all BACON_2 domain‐containing proteins are indeed frequently membrane‐bound and could likely provide local proximity of their varying C‐termini to the outer cell membrane (Fig. 3, Table S4).

### Structural evidence that DUF1735 proteins act as spacer

In the RCSB Protein Data Bank (PDB) the crystal structures of six proteins containing a DUF1735 domain and two proteins containing a BACON and/or BACON_2 domain were deposited (Table 4). DUF1735‐ and BACON domain‐containing proteins both have an immunoglobulin (Ig)‐like β‐sandwich fold with a core structure of two packed antiparallel β‐sheets. The Ig‐like β‐sandwich fold had been reported for the N‐termini of SusE and SusF of the Sus that also function as flexible spacer for their C‐terminal CBMs similar to what has been described for BACON [1, 25, 58]. However, it is important to mention that when comparing the Ig‐like β‐sandwich folds more closely differences become apparent (Fig. S2).

**Table 4:** Overview of all crystal structures of DUF1735‐, BACON‐, and BACON_2‐containing proteins. Data accessed in February 2024. + indicates separate domains, I indicates overlapping domains.

Domain architecture	GeneID	PDB ID	(predicted) function	PUL ID	Substrate	References
DUF1735 + GH18	BT_3987	6T8I, 6T8K, 6T8L, 6TCV, 6TCW, 7NWF, 3POH	*endo*‐β‐*N*‐acetylglucosamidase	Literature‐derived PUL72	High‐mannose mammalian *N*‐glycan (HMNG)	[22]
DUF1735 + DUF5627	BACOVA_03430	3N91	PUF, does not bind tested xylooligosaccharides (XOS)	Literature‐derived PUL114	Xylan	[56]
DUF1735 + GBDL	BF3416	4JX0	PUF	Predicted PUL 43		/
DUF1735 + LamG3/ConA‐like domain	BACOVA_03559	4DQA	Putative carbohydrate binding protein	Literature‐derived PUL 77		[41]
DUF4973IDUF1735 + DUF4361	BT3507	4QNI	Auxiliary nutrient binding protein	Literature‐derived PUL 59		–
DUF4973IDUF1735 + DUF4361	BACOVA_03322	3SOT	Multimodular protein	Literature‐derived PUL 69		–
BACON + GH5	BACOVA_02653	3ZMR	*endo*‐xyloglucanase (BoGH5A)	Literature‐derived PUL113 (and 47)	Xyloglucan	[53]
GH115 + BACONIBACON_2 + GH115_C	Q21JW4_SACD2	4ZMH	Five‐domain GH115 α‐glucuronidase	–	(oligomers of) glucuronoxylan	[62]

To strengthen the proposed function as spacer and highlight that a function as carbohydrate binding domain is expected to be unlikely, conservation studies on sequential and structural level were performed and combined. Surface‐exposed aromatic residues are known to interact with cyclic monosaccharides via CH/π‐stacking [59, 60, 61]. Only a two aromatic residues are conserved in the DUF1735 homologs and when visualized in the crystal structure of BT3987 (PDB: 6T8i), none of them appear to be on the surface. This makes a function of DUF1735 as carbohydrate binding module unlikely. Furthermore, all DUF1735 crystal structures deposited in PDB were analyzed to predicted interactions of the protein with other proteins, DNA/RNA, lipids, ligands, and ions [32]. Our analysis revealed that DUF1735 does not show clear predicted interactions with either of the tested entities. Only some residues of DUF1735 from 4QNI (DUF1735 + DUF4361) and BT3986 (DUF1735 + LamG3) revealed predicted protein interactions (data not shown).

Based on the shared Ig‐like β‐sandwich fold of DUF1735, SusE, SusF, and BACON; the genetic localization of DUF1735 at the *susE*‐F‐like position in PULs, and the fact that all were predominantly present as multimodular proteins with varying C‐termini involved in carbohydrate metabolism, a similar function as N‐terminal spacer for various C‐termini for DUF1735 was predicted.

## Conclusion

Our generated datasets and performed analysis of DUF1735 present in PULDB, KEGG, and UniProt revealed several indications that DUF1735 likely acts as N‐terminal membrane‐bound spacer of SusE‐ or SusF‐like proteins linked to varying C‐termini involved in carbohydrate binding or degradation. The most frequent C‐termini being LamG3, F5/8‐typeC, and GH18. Further, it was confirmed that DUF1735‐containing proteins were exclusively encoded in PULs (97.8%) and in genomes of species of the Bacteroidota phylum (98%) with a maximum of 46 copies in the genome of *B. thetaiotaomicron* 7330. Our comparison of DUF1735‐, BACON‐, and BACON_2 domain‐containing proteins revealed both commonalities, such as their N‐terminal position in multimodular proteins with suggested function of DUF1735 and BACON as membrane‐bound spacer and domain repetition in genomes, and distinctions, notably the rather significant differences in the Ig‐like β‐sandwich fold, the presence of DUF1735 in PULs and its less variable C‐termini. Mutation screenings and further investigation of the partial functionally characterized multimeric DUF1735‐containing protein BT3986 (DUF1735 + LamG3), BT3987 (DUF1735 + GH18), BT2624, and BT3790 (both DUF1735 + DUF4361) could unravel whether DUF1735 is crucial for the function of their C‐terminus.

## Conflict of interest

The authors declare no conflict of interest.

### Peer review

The peer review history for this article is available at https://www.webofscience.com/api/gateway/wos/peer‐review/10.1002/2211‐5463.13816.

## Author contributions

LH was involved in conceptualizing, performed major data analysis, literature review and was leading author in writing the manuscript. SB‐L initiated research and was involved in conceptualizing, literature review and initial manuscript writing. LAG was involved in conceptualizing, data analysis, preparation of tables, and critical reviewing. She developed the domain analyzer and vicinity analyzer, performed all work done with them, and wrote according paragraphs in the method section. TP initiated the conserved residue analysis and their structural localization and provided the cartoon representation of the three target domains. EJ was involved in conceptualizing, and critical reviewing the manuscript. All authors have approved the final article.

## Supporting information


**Fig. S1.** Output from PeSTo analysis of the alphafold model of BT3986 (DUF1735 + LamG) for surface ligand interactions.

**Fig. S2.** Cartoon representation of DUF1735 (PDB: 6T8i), BACON (PDB: 3ZMR), and BACON_2 (PDB: 4ZMH).

**Table S1.** Dataset of DUF1735‐containing proteins in literature‐derived PULs from PULDB.

**Table S2.** (A) Dataset of DUF1735 generated by mining KEGG with domain analyzer. (B) Dataset of BACON generated by mining KEGG with domain analyzer. (C) Dataset of BACON_2 generated by mining KEGG with domain analyzer.

**Table S3.** Datasets of DUF1735 generated by mining UniProt with domain analyzer.

**Table S4.** Investigation of genomic neighbors of DUF1735, BACON, and BACON_2 domain‐containing proteins using vicinity analyzer.

**Table S5.** Signal peptide prediction of DUF1735, BACON, and BACON_2 domain homologs.

**Table S6.** Domain architecture of DUF4973‐, DUF4361‐, and DUF5627‐containing proteins in KEGG based on data accessed in February 2024 [24].

## Data Availability

All data generated and analyzed during this study are included in this published article and its supplementary files.
