# Supplementary material for: Polysaccharide utilization loci encoded DUF1735 likely functions as membrane‐bound spacer for carbohydrate active enzymes
Source: FEBS Open Bio. 2024 May 12;14(7):1133–46. doi: 10.1002/2211-5463.13816 (PMC11216935; doi:10.1002/2211-5463.13816)
Supplement: Supplementary file 1 — Fig. S1. Output from PeSTo analysis of the alphafold model of BT3986 (DUF1735 + LamG) for surface ligand interactions. [file FEB4-14-1133-s004.docx]

AlphaFold Modeling of BT3986

For Figure 3 an AlphaFold model of BT3986 (DUF1735 + LamG3) from *Bacteroides thetaiotaomicron* (UniProt: Q8A0N5_BACTN) was generated using AlphaFold and ColabFold available at https://github.com/sokrypton/ColabFold [1,2]. The default settings num_relax: 0 and template_mode: none were used. The predicted structural model showed high per residue confidence scores (pLDDT): 86.6 for the DUF1735 domain (residues 26-166) and 94.3 for the LamG3 domain (residues 167-384), and was visualized in PyMOL2 [3].

Analysis of protein structures


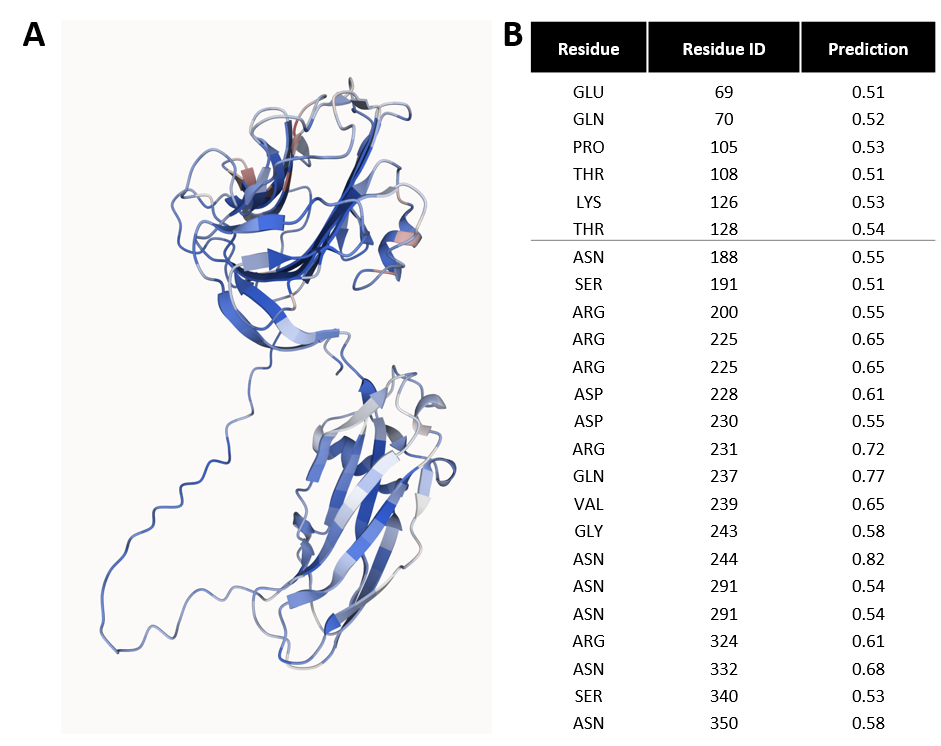


**Figure S1** Output from PeSTo (https://pesto.epfl.ch/) analysis of the AlphaFold model of BT3986 (DUF1735 + LamG) for surface ligand interactions [4]. **A)** Cartoon representation of the AlphaFold model color coded from light blue = no interactions to red = strong interactions. DUF1735 represented at the bottom, LamG at the top. **B)** Overview of residues with predicted interactions with ligands. InterPro analysis predicts DUF1735 from residue ID 32-149 and LamG3 187-330 [5].

References

1 Jumper J, Evans R, Pritzel A, Green T, Figurnov M, Ronneberger O, Tunyasuvunakool K, Bates R, Žídek A, Potapenko A, Bridgland A, Meyer C, Kohl SAA, Ballard AJ, Cowie A, Romera-Paredes B, Nikolov S, Jain R, Adler J, Back T, Petersen S, Reiman D, Clancy E, Zielinski M, Steinegger M, Pacholska M, Berghammer T, Bodenstein S, Silver D, Vinyals O, Senior AW, Kavukcuoglu K, Kohli P & Hassabis D (2021) Highly accurate protein structure prediction with AlphaFold. *Nature* **596**, 583–589.

2 Mirdita M, Ovchinnikov S, Steinegger M, Schütze K, Moriwaki Y & Heo L (2022) ColabFold: Making protein folding accessible to all. *Nat Methods* **19**, 679–682.

3 Schrödinger & LLC (2015) The PyMOL molecular graphics system. .

4 Krapp LF, Abriata LA, Cortés Rodriguez F & Dal Peraro M (2023) PeSTo: parameter-free geometric deep learning for accurate prediction of protein binding interfaces. *Nature Communication* **14**.

5 Paysan-Lafosse T, Blum M, Chuguransky S, Grego T, Azaro Pinto BL´, Salazar GA, Bileschi ML, Bridge A, Colwell L, Gough J, Haft DH, Letuní I, Marchler-Bauer A, Mi H, Natale DA, Orengo CA, Pandurangan AP, Rivoire C, Sigrist CJA, Sillitoe I, Thanki N, Thomas PD, Tosatto SCE, Wu CH & Bateman A (2023) InterPro in 2022. *Nucleic Acids Res* **51**, D418–D427.
