# Supplementary figures and images for: Polysaccharide utilization loci encoded DUF1735 likely functions as membrane‐bound spacer for carbohydrate active enzymes
Source: FEBS Open Bio. 2024 May 12;14(7):1133–46. doi: 10.1002/2211-5463.13816 (PMC11216935; doi:10.1002/2211-5463.13816)

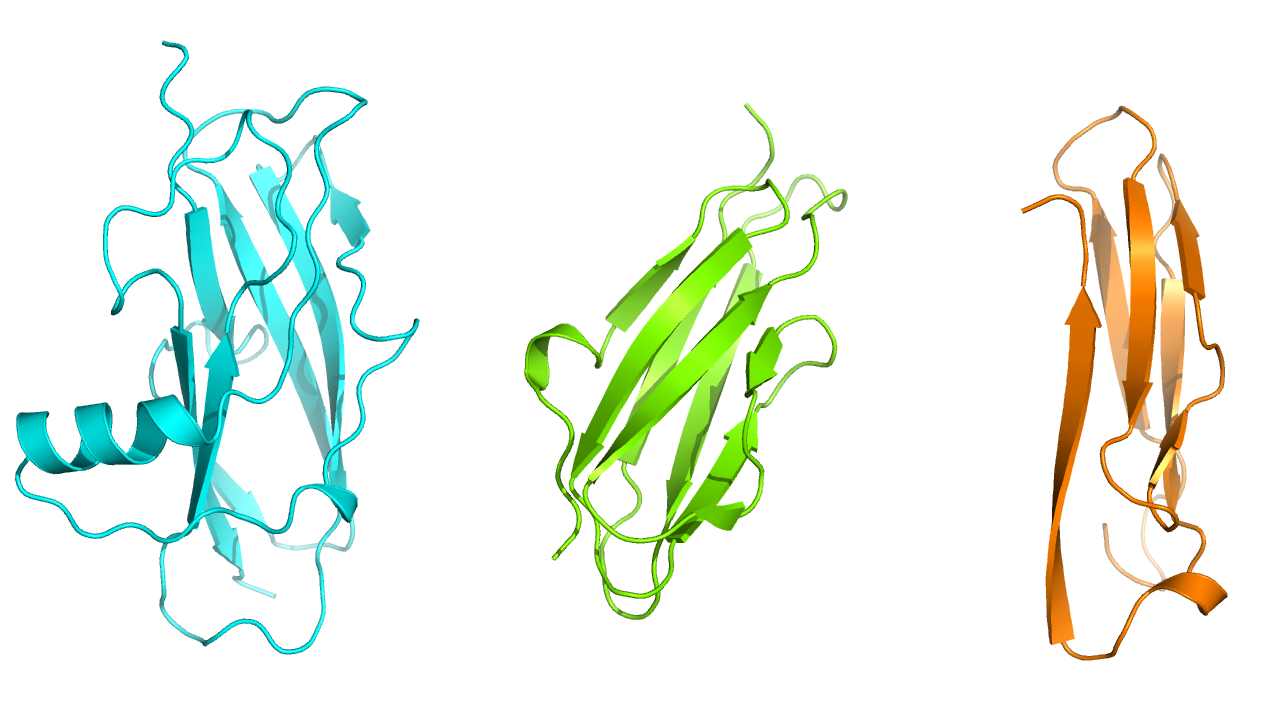


**Figure S2** Cartoon representation of DUF1735 (PDB: 6T8i, left), BACON (PDB: 3ZMR, middle), and BACON_2 (PDB: 4ZMH, right).

Supplement: Supplementary file 2 — Fig. S2. Cartoon representation of DUF1735 (PDB: 6T8i), BACON (PDB: 3ZMR), and BACON_2 (PDB: 4ZMH). [file FEB4-14-1133-s006.docx]
