# Supplementary material for: Polysaccharide utilization loci encoded DUF1735 likely functions as membrane‐bound spacer for carbohydrate active enzymes
Source: FEBS Open Bio. 2024 May 12;14(7):1133–46. doi: 10.1002/2211-5463.13816 (PMC11216935; doi:10.1002/2211-5463.13816)
Supplement: Supplementary file 7 — Table S5. Signal peptide prediction of DUF1735, BACON, and BACON_2 domain homologs. [file FEB4-14-1133-s003.docx]

**Table S5** Signal peptide prediction with SignalP6.0 [1] for protein sequences of DUF1735 (4047), BACON (5942), and BACON_2 domain homologs (4515) in UniProt [2].

| **Type of predicted signal peptide** | **DUF1735** | | **BACON** | | **BACON_2** | |
| --- | --- | --- | --- | --- | --- | --- |
|  | **[n]** | **[%]** | **[n]** | **[%]** | **[n]** | **[%]** |
| Lipoprotein signal peptides (Sec/SPII) -LIPO | 3875 | 96 | 4545 | 76 | 1448 | 32 |
| Tat lipoprotein signal peptides (Tat/SPII) - TATLIPO | 1 | 0 | - | - | - | - |
| Secretory signal peptides (Sec/SPI) - SP | 35 | 1 | 574 | 10 | 1121 | 25 |
| OTHER | 136 | 3 | 823 | 14 | 1934 | 43 |

1 The Uniprot Consortium (2017) UniProt: the universal protein knowledgebase. *Nucleic Acids Res* **45**, D158–D169.

2 Teufel F, Almagro Armenteros JJ, Johansen AR, Gíslason MH, Pihl SI, Tsirigos KD, Winther O, Brunak S, von Heijne G & Nielsen H (2022) SignalP 6.0 predicts all five types of signal peptides using protein language models. *Nat Biotechnol* **40**, 1023–1025.
