# Supplementary material for: Polysaccharide utilization loci encoded DUF1735 likely functions as membrane‐bound spacer for carbohydrate active enzymes
Source: FEBS Open Bio. 2024 May 12;14(7):1133–46. doi: 10.1002/2211-5463.13816 (PMC11216935; doi:10.1002/2211-5463.13816)
Supplement: Supplementary file 8 — Table S6. Domain architecture of DUF4973‐, DUF4361‐, and DUF5627‐containing proteins in KEGG based on data accessed in February 2024 [24]. [file FEB4-14-1133-s001.docx]

**Table S6** Domain architecture of DUF4973-, DUF4361-, and DUF5627-containing proteins in KEGG based on data accessed in February 2024 [1]. + indicates separate domains, I indicates overlapping domains.

| **DUF4973** (n=154) | **[n]** | **[%]** |
| --- | --- | --- |
| DUF4973\|DUF1735 + DUF4361 | 72 | 46.8% |
| DUF1735\|DUF4973 + DUF4361 | 36 | 23.4% |
| DUF1735\|DUF4973 | 19 | 12.3% |
| DUF1735\|DUF4973 + LamG3 | 7 | 4.5% |
| DUF4973 + DUF1735 + DUF4361 | 5 | 3.2% |
| DUF1735\|DUF4973 + F5/8-typeC | 4 | 2.6% |
| DUF4973\|DUF1735 | 4 | 2.6% |
| DUF1735\|DUF4973 + DUF5627 | 2 | 1.3% |
| DUF1735\|DUF4973 + DUF1735 + F5/8-typeC | 1 | 0.6% |
| DUF1735\|DUF4973 + F5/8-typeC + Sad1_UNC | 1 | 0.6% |
| DUF4973 + DUF4361 | 1 | 0.6% |
| DUF4973\|DUF1735 + DUF1735 + DUF4361 | 1 | 0.6% |
| DUF4973\|DUF1735 + F5/8-typeC | 1 | 0.6% |
| **DUF4361** (n=149) | **[n]** | **[%]** |
| DUF4973\|DUF1735 + DUF4361 | 78 | 52.3% |
| DUF1735\|DUF4973 + DUF4361 | 36 | 24.2% |
| DUF1735 + DUF4361 | 32 | 21.5% |
| DUF1735 + DUF5627\|DUF4361 | 1 | 0.7% |
| DUF4361 | 1 | 0.7% |
| DUF4973 + DUF4361 | 1 | 0.7% |
| **DUF5627** (n = 76) | **[n]** | **[%]** |
| DUF1735 + DUF5627 | 73 | 96.1% |
| DUF1735\|DUF4973 + DUF5627 | 2 | 2.6% |
| DUF1735 + DUF4361\|DUF5627 | 1 | 1.3% |

1 Kanehisa M & Goto S (2000) KEGG: Kyoto Encyclopedia of Genes and Genomes. *Nucleic Acids Res* **28**, 27–30.
